# Ozone

**Published:** 1879-03

**Authors:** 


OZONE, which was discovered by SCHONBEIN within 25 years ; he gave it the name of Ozone, which means smell, he having first ascertained its presence in his laboratory by its odor, which is something like that of phosphorus, noticeable sometimes, immediately after very loud thunder and vivid lightning. SCHONBEIN had observed the same odor some years before, in 1840, at the positive pole of a platinum battery, used in decomposing water by electricity, and the same afterwards in the slow combustion of phosphorus and ether.

The presence of Ozone in the atmosphere may be ascertained thus : Starch a piece of soft paper or muslin and dip it into a solution of iodide of potash, then expose it to the air ; it turns brown first, and, when moistened, gives various shades of a pinkish blue, more or less deep, according to the greater amount of ozone contained in the air ; a scale has been made of ten degrees, showing the relative amounts of ozone, with the accuracy of a thermometer measuring the degree of heat or cold. The principal European chemists consider that a more accurate and sensitive test of the presence of Ozone in the atmosphere is made by saturating strips of paper with the tincture of guiacum.

If the air contains one fifty thousandth part of ozone, its odor is perceived, and yet so delicate is the test of the scale, that four degrees lower, its presence is manifest. If one five thousandth part of ozone is in the air breathed, insects die, by being consumed—burned up ; for as oxygen is the great oxydyzer in the universe, and Ozone being an electric or more powerful or concentrated form of oxygen with greater freedom and perfectness, it may be considered the fire of fire to all insect life. Although so little ozone in the atmosphere is required for health, yet, as this proportion diminishes, epidemic diseases are sure to appear ; this has been repeatedly noticed by scientific men, from which the inference may be safely drawn, that the less ozone there is in the air, the more full it is of that kind of life which corrupts the blood ; while the larger presence of ozone destroys this life, and we say "the air is so pure and lovely."

